# Management and Outcome of COVID-19 Positive and Negative Patients in French Emergency Departments During the First COVID-19 Outbreak: A Prospective Controlled Cohort Study

**DOI:** 10.5811/westjem.2022.7.57135

**Published:** 2022-10-24

**Authors:** Marion Douplat, Antoine Gavoille, Fabien Subtil, Julie Haesebaert, Laurent Jacquin, Guillaume Durand, Jean-Christophe Lega, Thomas Perpoint, Veronique Potinet, Julien Berthiller, Nathalie Perreton, Karim Tazarourte

**Affiliations:** * Université Claude Bernard Lyon 1, Research on Healthcare Performance (RESHAPE), INSERM U1290, Lyon, France; † Université de Lyon Université Lyon 1, CNRS, Laboratoire de Biométrie et Biologie Évolutive UMR 5558, Villeurbanne, France; ‡ Hospices Civils de Lyon, Edouard Herriot Hospital, Department of Emergency Medicine, Lyon, France; § Villefranche Hospital, Department of Emergency Medicine, Gleize, France; ¶ Hospices Civils de Lyon, Lyon Sud Hospital, Department of Internal and VascularMedicine, Pierre Bénite, France; || Service de Maladies Infectieuses et Tropicales, Hôpital Croix-Rousse Hospices Civils de Lyon, Lyon, France; # Hospices Civils de Lyon, Lyon Sud Hospital, Department of Emergency Medicine, Pierre Bénite, France; ** Service de Biostatistique, Hospices Civils de Lyon, Lyon France; †† Pôle de Santé Publique, Service de Recherche et d’Epidémiologie Cliniques, Hospices Civils de Lyon, France

## Abstract

**Introduction:**

Few studies have investigated the management of COVID-19 cases from the operational perspective of the emergency department (ED), We sought to compare the management and outcome of COVID-19 positive and negative patients who presented to French EDs.

**Methods:**

We conducted a prospective, multicenter, observational study in four EDs. Included in the study were adult patients (≥18 years) between March 6–May 10, 2020, were hospitalized, and whose presenting symptoms were evocative of COVID-19. We compared the clinical features, management, and prognosis of patients according to their confirmed COVID-19 status.

**Results:**

Of the 2,686 patients included in this study, 760 (28.3%) were COVID-19 positive. Among them, 364 (48.0%) had hypertension, 228 (30.0%) had chronic cardiac disease, 186 (24.5%) had diabetes, 126 (16.6%) were obese, and 114 (15.0%) had chronic respiratory disease. The proportion of patients admitted to intensive care units (ICU) was higher among COVID-19 positive patients (185/760, 24.3%) compared to COVID-19 negative patients (206/1,926, 10.7%; P <0.001), and they required mechanical ventilation (89, 11.9% vs 37, 1.9%; P <0.001) and high-flow nasal cannula oxygen therapy (135, 18.1% vs 41, 2.2%; P < 0.001) more frequently. The in-hospital mortality was significantly higher among COVID-19 positive patients (139, 18.3% vs 149, 7.7%; P <0.001).

**Conclusion:**

Emergency departments were on the frontline during the COVID-19 pandemic and had to manage potential COVID-19 patients. Understanding what happened in the ED during this first outbreak is crucial to underline the importance of flexible organizations that can quickly adapt the bed capacities to the incoming flow of COVID-19 positive patients.

## INTRODUCTION

The coronavirus disease 2019 (COVID-19) pandemic was declared on March 11, 2020, by the World Health Organization.^1^–^3^ From December 31, 2019–January 2021, 98,280,844 cases were confirmed worldwide, among which 32,848,998 were in Europe.^4^ France was one of the countries most impacted by the COVID-19 pandemic, with 3,130,629 confirmed cases and 74,800 deaths during this period.^5^ The first outbreak started in France at the beginning of March 2020, and containment was officially established from March 17–May 11, 2020.^5^

French emergency departments (ED) were on the frontline during the COVID-19 outbreak and oversaw patient triage, based on COVID-19 suspicion, as they were in other countries.^6^,^7^ The role of the ED in patient triage was crucial to contain and isolate the suspected COVID-19 cases. The need for a dynamic in patient flow processing has been highlighted,^8^ and several hospital emergency management plans have been proposed, including a before-admission triage center.^9^–^11^ Several studies have focused on the outcomes of patients during the COVID-19 pandemic, but few have investigated the management of COVID-19 cases from the perspective of EDs.^12^–^14^ However, the need to understand how to manage these patients in EDs is necessary to avoid crowding, guarantee the safety of healthcare workers, anticipate the future need for beds and staff members, and to be able to continue caring for non-COVID-19 patients.^12^,^15^

As the number of COVID-19 cases was rapidly increasing in France at the beginning of March 2020 we set up the COVID-ER cohort study. Our goal was to provide an exhaustive description over time of the management and outcome of patients presenting to French EDs for COVID-19 suspicion from March–May 2020 and to determine whether they were different depending on the patients’ COVID-19 status. We describe the characteristics associated with COVID-19 diagnosis confirmation and prognosis, including admission to the intensive care unit (ICU) and all-cause mortality.

## METHODS

### Study Design and Setting

We conducted a multicenter prospective observational cohort study March 6–May 10, 2020 in four French EDs within three university hospitals (*Hôpital Edouard Herriot*, *Centre Hospitalier Lyon Sud*, and *Hôpital de la Croix-Rousse*) and one general hospital (*Hôpital de Villefranche*) in and around Lyon. The Lyon urban area is the second largest in France with a population of 1.6 million. The three university EDs are in urban hospitals: two of them receive more than 40,000 ED visits per year, while the third has 80,000 visits annually. The ED of the general hospital is suburban and has 50,000 ED visits per year. This study complied with the Declaration of Helsinski, and was approved by both the institutional ethics committee of the *Hospices Civils of Lyon* (number [n°] 20–47) and the *Comission Nationale de l’Informatique et des Libertés* (CNIL, French commission for data protection; n° 20–090), as required by French law. This paper complies with the STROBE guidelines for reporting observational studies.^16^ Per French legislation, only oral consent was required. This was approved by the ethics committee of the *Hospices Civils of Lyon* (20–47) and the CNIL (n° 20–090). All patients were informed that their data was being collected as part of the COVID-ER study via written notice and had the opportunity to object to the collection of their information.

**Population Health Research Capsule**

What do we already know about this issue?

*Emergency departments were on the frontline during the COVID-19 outbreak and oversaw patient triage*.

What was the research question?

*We sought to determine whether the management of patients presenting to French EDs for suspected COVID-19 was different depending on their COVID-19 status*

What was the major finding of the study?

*Patients admitted to intensive care units was higher among COVID-19 positive (24.3%) vs negative patients (10.7%; P <0.001)*.

How does this improve population health?

*Our findings underline the importance of organizational flexibility to quickly adapt hospital capacities to the surge of COVID-19 positive patients into EDs*

### Selection of Participants

We included in the study all adult patients (≥18 years) presenting to the ED for suspected COVID-19 (with symptoms evocative of severe acute respiratory syndrome coronavirus 2 [SARS-CoV-2]) infection and requiring hospitalization. We classified the clinical presentation of suspected COVID-19 patients according to their level of severity: level 1 represented the most critical patients, who were initially managed in the ED and then admitted to the ICU for intubation; levels 2 and 3 were managed in the ED. Level 4 cases met none of the criteria for severity when compared to levels 1–3; hence, they were not managed in the ED and were sent home with medical advice (Supplementary Figure S1). Healthcare workers who were infected did not go to work and were managed by the occupational health service of each hospital. However, if they were in respiratory distress, they could present to the ED.

We excluded patients without symptoms of SARS-CoV-2 infection, as well as patients with another confirmed infectious diagnosis in the ED such as intra-abdominal, skin and soft tissue infection, or genital and urinary tract infection, and those with suspected meningitis. Also excluded were COVID-19-suspected patients who did not require hospitalization and were sent home without testing, due to the limited availability of SARS-CoV-2-specific reverse transcriptase polymerase chain reaction (RT-PCR) tests in France at the time of the study.

Patients were tested for SARS-CoV-2 infection using RT-PCR on respiratory samples. The RT-PCR assays were performed using the RdRp IP2-IP4 primers and probes per *Institut Pasteur* protocol, which is used in France for SARS-CoV-2 detection. This protocol, detecting two targets in the *RdRp* gene, was adapted on the Panther Fusion molecular system for high throughput diagnostics (Hologic Inc, Marlborough, MA). A confirmed case of COVID-19 was defined as a SARS-CoV-2-specific positive RT-PCR test. In cases of multiple sampling during hospitalization, we classified the final virological diagnostic as positive if one of the samples had tested positive. We compared the management and outcome between COVID-19 positive and negative patients among the population included.

### Data Collection and Processing

We collected the following data for each patient from electronic health records: demographic characteristics (age, gender, place of residence, functional independence, healthcare worker status); and clinical characteristics (symptoms and vital signs at ED admission, size, weight, chronic underlying comorbidities, smoking status). The chronic underlying diseases considered were as follows: hypertension; diabetes; clinical heart failure (NYHA functional class III or IV), obesity (body mass index [BMI]≥30 kilograms per meter squared); chronic respiratory disease defined as chronic restrictive or obstructive pulmonary disease; chronic kidney disease (glomerular filtration rate <90 milliliters per minute); chronic neurological disorder; chronic hematological disease; immunosuppression; transplant; cirrhosis; dementia (if it had been documented by a Mini-Mental State Examination score under 24); malignancy (defined as current malignancy with or without metastasis); psychosis; and human immunodeficiency virus infection. We also collected laboratory findings (other viral and bacterial infection) and radiology findings (chest computed tomography [CT]). A CT was considered positive for COVID-19 if there were features evocative of COVID-19: ground-glass opacity; crazy-paving pattern; sub-pleural bands of consolidations, reversed halo sign; and lung consolidations.

We collected the vital signs recorded in the ED and during hospitalization for the whole cohort. We also collected patient management data: admission from the ED to the ICU or conventional hospitalization, secondary admission from conventional hospitalization to the ICU; ventilation support; decision to withhold or withdraw life-sustaining treatments; and re-hospitalization within 30 days after discharge.

### Primary Data Analysis

Continuous variables were expressed as mean ± SD, or median (interquartile range [IQR]) for duration, and categorical variables as count (percentage). We compared the characteristics of COVID-19 positive and COVID-19 negative patients using chi square and Fisher’s exact tests, or the Wilcoxon rank-sum test. Comparisons of outcomes between the COVID-19 positive and COVID-19 negative groups were performed using logistic regression for binary outcomes and using linear regression with logarithmic transformation for delays.

We performed multivariate analyses to take into account putative confounding factors. Adjustments were performed on factors that displayed the greatest imbalance between COVID-19 positive and negative patients, except factors related to the condition at admission, and that were associated with most of the different outcomes in univariate analyses. The effect of COVID-19 status on the outcomes was adjusted for age, gender, BMI, smoking status, loss of autonomy (correlated with the place of residence), chronic respiratory disease, malignancy, bacterial infection, and oxygen requirement. The viral infection status was not included in multivariate analyses due to multicolinearities. Unless specified otherwise, the *P*-values reported corresponded to the ones of multivariate analyses. *P*-values were considered significant below 0.05. We performed analyses using R, version 3.6.1. (R Core Team [2019], Vienna, Austria, https://www.R-project.org/).

## RESULTS

**Figure 1:**
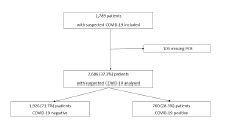
Trial profile of patients admitted to emergency departments during the study period. *COVID-19*, coronavirus disease 2019; *PCR*, polymerase chain reaction.

From March 6–May 10, 2020, 20,341 patients presented to the participating EDs, of whom 7,199 (35.4%) were hospitalized and 2,789 were suspected of SARS-CoV-2 infection. A total of 2,686 patients were eventually included in our study (1,926 COVID-19 positive patients and 760 COVID-19 negative patients (Figure 1).

### Patient Characteristics According to COVID-19 Status

The mean ± SD age of COVID-19 positive patients was 71.5 ± 16.5 years, of whom 618 (81.6%) presented from home and 119 (15.7%) from long-term care facilities. A total of 395 (52.1%) COVID-19 positive patients were referred by emergency medical services. Hypertension was present in 364 (48.0%) COVID-19 positive patients; chronic cardiac disease in 228 (30.0%); diabetes in 186 (24.5%); obesity in 126 (16.6%); and chronic respiratory disease in 114 (15.0%) (Table 1).

Oxygen was required upon arrival at the EDs for 179 (23.6%) COVID-19 positive patients, and for 134 (18.3%) COVID-19 negative patients. A total of 215 (30.6%) COVID-19 positive patients presented to the EDs more than seven days after symptom onset while 315 (19.0%) COVID-19 negative patients did, and 105 (15.0%) COVID-19 positive patients presented during the first 24 hours after symptom onset (while 613 (36.9%) COVID-19 negative patients did). Fever was encountered in 536 (70.5%) COVID-19 positive patients, dyspnea in 494 (65.0%), cough in 420 (55.3%), weakness in 399 (52.5%), and anosmia in 51 (6.7%). Bacterial infection was found in 57 (9.3%) COVID-19 positive patients and co-viral infection in eight (2.5%). A total of 454 (59.7%) COVID-19 positive patients had a CT evocative of COVID-19, while 237 (12.3%) COVID-19 negative patients did (Table 1).

### ICU Admission and Ventilation Support

A total of 185 (24.3%) COVID-19 positive patients were admitted to the ICU while 206 (10.7%) COVID-19 negative patients were admitted (odds ratio [OR] 2.24 [1.57; 3.20]; *P* <0.001). The proportion of patients secondarily admitted to the ICU was also higher among COVID-19 positive patients compared to COVID-19 negative patients (OR 5.90 [3.47; 10.24]; *P* <0.001). Invasive mechanical ventilation and high-flow nasal cannula oxygen therapy were more often used for COVID-19 positive than negative patients (OR 6.82 [3.87, 12.42]; *P* <0.001, and OR 10.08 [5.89, 17.87]; *P* <0.001, respectively (Table 2).

### Conventional Hospitalization

**Figure 2:**
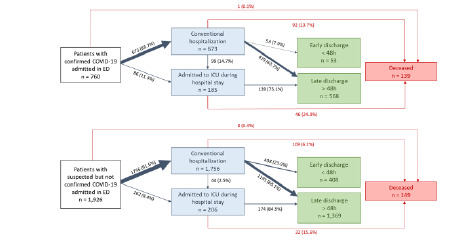
COVID-19 positive and COVID-19 negative patients’ management. *ED*, emergency department; *ICU*, intensive care unit; *COVID-19*, coronavirus disease 2019

The number of conventional hospitalizations was higher among COVID-19 negative patients compared to COVID-19 positive patients (*P* = 0.036; Table 2). Among the 673 COVID-19 positive patients who were conventionally hospitalized, 53 (7.9%) were discharged early (<48 hours) from the hospital, while 408 (23.9%) COVID-19 negative patients were discharged early (Figure 2).

### Mortality and Decisions to Withhold or Withdraw Life-sustaining Treatments

Mortality during hospitalization was significantly higher among COVID-19 positive patients compared to COVID-19 negative patients (OR 3.33, [2.02, 5.50]; *P* <0.001). Among the 185 COVID-19 positive patients who were admitted to the ICU, 46 (24.9%) died, compared to 32/206 (15.6%) ICU-admitted COVID-19-negative patients. Among the 673 COVID-19 positive patients who were conventionally hospitalized, 92 (9.7%) died, compared to 109/1,756 (6.2%) COVID-19 negative patients (Table 2). Only one (0.1%) COVID-19 positive patient compared to eight (0.4%) COVID-19 negative patients died in the ED (Figure 2). The number of decisions to withhold or withdraw life-sustaining treatments was higher during hospitalization concerning COVID-19 positive patients than COVID-19 negative patients (OR 2.08 [1.31, 3.28]; *P =* 0.002), and there was no significant difference in EDs (OR 1.81 [0.85, 3.72], *P* = 0.113 (Table 2).

### Hospital Discharge

The median [IQR] length of stay in hospital was significantly longer for COVID-19 positive patients (10 [6–15] days) compared to COVID-19 negative patients (6 [2–11] days; *P* <0.001). After hospital discharge, a greater proportion of COVID-19 positive patients were admitted into a rehabilitation department before returning home (157/554, 28.3%) compared to COVID-19 negative patients (245/1627, 15.1%; *P* <0.001 (Table 2).

### Factors Associated with ICU Admission and Mortality

The ICU admission rate was higher for patients with a positive COVID-19 status (*P* <0.001); oxygen requirement (*P* <0.001); male gender (*P* <0.001), and lower with increasing age (*P* <0.001) and malignancy (*P* <0.001) in multivariate analysis (Table 3). The mortality risk was higher with a positive COVID-19 status (*P* <0.001), for men (*P* = 0.006); malignancy (*P* = 0.039); oxygen requirement (*P* <0.001); bacterial infection (*P* <0.001); and with increasing age (*P* <0.001) in multivariate analysis (Table 4).

## DISCUSSION

The study cohort was composed of a large sample of patients admitted to the ED for suspected COVID-19 over a period that included the totality of the first containment in France. The region of Lyon was one of the most impacted during the first outbreak, after the *Grand Est* region and the *Île-de-France* region, including Paris, which provided an interesting viewpoint regarding the management of the COVID-19 pandemic in EDs.

We found that among the patients presenting to EDs with suspected COVID-19, those who were actually COVID-19 positive were more often admitted to the ICU than were conventionally hospitalized, required more invasive mechanical ventilation, and stayed longer in the hospital compared to COVID-19 negative patients. The results presented herein also suggested that among COVID-19 suspected patients, factors such as positive COVID-19 status, oxygen requirement, and male gender were at risk for ICU admission and mortality. Mortality also increased with age, malignancy, and bacterial infection.

The characteristics of the COVID-19 positive patients in our study broadly reflect those reported in other studies, especially in terms of symptoms and comorbidities.^2^,^3^;^17^–^19^ The rate of obesity was low, about two times lower than in the United States of America (US). These trends are consistent with the prevalence of obesity in the general population in France and the US.^20^ COVID-19 positive patients had a higher median age than patients in China,^2^ the US,^7^ and Italy,^18^ but a similar median age compared to patients in the United Kingdom (UK).^19^ These differences may be explained by the different recruitment methods that were used. We did not include ambulatory patients, who are most often younger, but we did include all hospitalized patients (corresponding to older patients who are more vulnerable and frail).

The proportion of COVID-19 positive patients admitted to the ICU was higher compared to previous studies conducted in the US (New York)^12^,^18^ and the UK.^19^ Several factors may explain these differences. First, the availability of ICU beds is different between countries. At the time of this study, the ICUs in our study were not overloaded but still reached maximum capacities despite a 30% increase in the number of beds during the first COVID-19 outbreak. Second, we included secondary ICU admissions in the follow-up, which were more numerous than primary admissions (unlike in the previously mentioned studies where they were not always considered). They correspond to patients who worsened secondarily within an average of 1–2 days. This point was also made by Singer et al who emphasized the need to take secondary ICU admissions into account to better estimate ICU capacities. Indeed, they demonstrated that for every 100 persons under investigation who are admitted to the hospital, nine will require immediate ICU admission and another 12 will require ICU or invasive mechanical ventilation within 2-3 days.^12^ Finally, the use of mechanical ventilation for COVID-19 positive patients was similar to its use in other studies.^18^,^19^ whereas the rates of high-flow nasal cannula oxygen therapy and non-invasive ventilation were higher in our study, suggesting that practices differ across countries.^22^

The mortality rate observed herein was lower compared to the one reported in the UK population,^19^ but not different from the one reported in the US^18^,^21^ or in Italy.^17^ This could be due to differences in healthcare systems between the UK and Europe and in the proportion of ICU beds to hospital beds, as previously suggested.^19^ In addition, patient comorbidities and drug exposure (including glucocorticoids) may differ between cohorts.

The decisions to withhold and withdraw life-sustaining treatments during the COVID-19 pandemic have been rarely studied due to the difficulty of collecting data regarding the a priori-decided level of care.^19^ In the current study, we report a high prevalence of these decisions concerning COVID-19 positive patients. However, there was no difference in the number of these decisions prior to death between COVID-19 positive and negative patients. We believe this can be explained by the fact that the COVID-19 health crisis led healthcare teams to anticipate the potential aggravation of a patient’s condition. Indeed, it has been previously shown that there was little anticipation regarding end-of-life decisions in the ED and that the management of such decisions should be improved.^23^,^24^ The decision-making process is especially difficult in the context of emergency medicine due to lack of time, absence of anticipation in treating chronic diseases, and restrictions of access to families as a result of the pandemic. Therefore, the healthcare teams faced several challenges with these decisions for which the consequences have not been well assessed.^25^

Understanding what happened during this first outbreak in the EDs included in this study is crucial to anticipate other health crises. Emergency departments are on the frontline during this type of crisis and must also manage potential COVID-19 patients, which contributes to the healthcare burden and ED crowding. In Australia, despite the low rate of COVID-19 positive cases, an increasing number of ED patients are likely to require isolation because the testing criteria have been broadened.^26^ The same has been reported in New York EDs where more than two thirds of all the admissions were patients suspected of COVID-19.^12^

## LIMITATIONS

This study has several limitations. First, we included primarily university hospitals, which have a greater ICU capacity; this certainly influenced the ICU admission rate. Second, the study was conducted only during the first outbreak and over a reduced period. Since then, practices have changed: the test criteria are broader; corticosteroids (mainly dexamethasone) have been introduced systematically for the most critical patients; and there has been an increase in physician expertise. Thirdly, the baseline comparison group could have been made up of patients admitted to the EDs prior to the COVID-19 outbreak in order to estimate the impact of the outbreak on the EDs; nevertheless, comparing patients admitted for COVID-19 suspicion and with a similar severity (probably only the most severe patients actually came to the EDs during the first lockdown) allowed us to limit the discrepancies in terms of baseline characteristics between groups. We probably had some false negatives especially during early phases of testing. Moreover, we did not initially include gastrointestinal symptoms as a presentation given the limited knowledge of COVID-19 at the beginning of the pandemic. Finally, despite the use of a multivariable model, we could not exclude residual confounders.

## CONCLUSION

This first outbreak of COVID-19 helped us to better quantify the need for ICU beds and to underline the importance of flexible organization to quickly adapt conventional and ICU capacities to the incoming flow into EDs of COVID-19 positive patients.

## Supplementary Information



## Figures and Tables

**Table 1:** Clinical, radiological, and laboratory characteristics of patients according to their COVID-19 status.

Characteristics	COVID-19 negative patients (n = 1,926, 71.7%)	COVID-19 positive patients (n = 760, 28.3%)	*P*
Age (years)	70.8 ± 18.6	71.5 ± 16.5	0.731
Female gender	976 (50.7%)	330 (43.4%)	<0.001
Living place (n = 2,653)			0.014
Home	1,579 (83.3%)	618 (81.6%)	
Long-term care facilities	226 (11.9%)	119 (15.7%)	
Other hospital	54 (2.8%)	11 (1.5%)	
Homeless	6 (0.3%)	1 (0.1%)	
Other	31 (1.6%)	8 (1.1%)	
Referred to ED by (n = 2,648)			<0.001
Emergency medical services	853 (45.1%)	395 (52.1%)	
General practitioners	497 (26.3%)	201 (26.5%)	
Individual decision	295 (15.6%)	86 (11.3%)	
Other	245 (13.0%)	76 (10.0%)	
Loss of autonomy	602 (31.3%)	196 (25.8%)	0.006
Healthcare worker (n = 2,558)	22 (1.2%)	17 (2.3%)	0.055
Current smoker (n = 2,002)	296 (20.1%)	36 (6.8%)	<0.001
BMI (n = 2,427)	25.79 ± 6.26	26.66 ± 5.54	<0.001
Comorbidities			
Hypertension	919 (47.8%)	364 (48.0%)	0.981
Chronic cardiac disease	696 (36.2%)	228 (30.0%)	0.003
Diabetes	471 (24.5%)	186 (24.5%)	1
Chronic respiratory disease	482 (25.1%)	114 (15.0%)	<0.001
Obesity	322 (16.7%)	126 (16.6%)	0.976
Chronic kidney disease	220 (11.5%)	70 (9.2%)	0.111
Immunosuppression	226 (11.8%)	28 (3.7%)	<0.001
Malignancy	203 (10.6%)	31 (4.1%)	<0.001
Dementia	132 (6.9%)	60 (7.9%)	0.392
Chronic neurological disorder	90 (4.7%)	34 (4.5%)	0.907
Chronic hematological disease	51 (2.7%)	7 (0.9%)	0.009
Cirrhosis	44 (2.3%)	10 (1.3%)	0.144
Psychosis	39 (2.0%)	11 (1.4%)	0.400
Transplant	22 (1.1%)	6 (0.8%)	0.547
HIV infection	11 (0.6%)	6 (0.8%)	0.590
Vital signs at ED admission			
Temperature (°C) (n = 2,627)	37.11 ± 1.07	37.58 ± 1.08	<0.001
Oxygen saturation (n = 2,620)	94.78 ± 4.67	92.62 ± 5.40	<0.001
Oxygen requirement	353 (18.3%)	179 (23.6%)	0.003
Time since symptom onset (n = 2,361)			<0.001
<24 hours	613 (36.9%)	105 (15.0%)	
<7 days	731 (44.1%)	382 (54.4%)	
<15 days	207 (12.5%)	181 (25.8%)	
≥15 days	108 (6.5%)	34 (4.8%)	
Symptoms (n from 2,669 to 2,686)			
Fever	916 (47.6%)	536 (70.5%)	<0.001
Dyspnea	1,036 (53.8%)	494 (65.0%)	<0.001
Cough	759 (39.5%)	420 (55.3%)	<0.001
Weakness	748 (38.8%)	399 (52.5%)	<0.001
Diarrhea	251 (13.1%)	168 (22.1%)	<0.001
Nausea or vomiting	339 (17.6%)	87 (11.4%)	<0.001
Myalgia	148 (7.7%)	84 (11.1%)	0.007
Headache	198 (10.3%)	86 (11.3%)	0.503
Confusion	198 (10.3%)	80 (10.5%)	0.926
Abdominal pain	339 (14.8%)	57 (7.5%)	<0.001
Anosmia	34 (1.8%)	51 (6.7%)	<0.001
Rhinorrhea/congestion	56 (2.9%)	26 (3.4%)	0.570
Sore throat	40 (2.1%)	10 (1.3%)	0.242
Joint pain	37 (1.9%)	11 (1.4%)	0.497
Bacterial infection (n = 2,126)	221 (14.6%)	57 (9.3%)	<0.001
Viral infection (n = 814)	34 (6.8%)	8 (2.5%)	0.011
Type of Viral infection			
Influenza A	13 (2.9%)	4 (1.3%)	<0.224
Influenza B	5 (1.1%)	3 (1.0%)	1
RSV	6 (1.4%)	4 (1.3%)	1
Rhinovirus	7 (5.7%)	0 (0.0%)	0.305
Metapneumovirus	3 (2.3%)	0 (0.0%)	0.748
Adenovirus respiratory	1 (0.8%)	1 (2.3%)	0.985
Positive CT chest (n = 1,686)			<0.001
Positive	237 (12.3%)	454 (59.7%)	
Negative	949 (49.3%)	46 (6.1%)	
Not done	740 (38.4%)	260 (34.2%)	

Data are expressed as count (percentage), or mean ± SD.

*COVID-19*, coronavirus disease 2019; *ED*, emergency department; *BMI*, body mass index *HIV*, human immunodeficiency virus.

**Table 2:** Outcomes of patients according to their COVID-19 status.

Outcomes	COVID-19 negative patients (n = 1,926)	COVID-19 positive patients (n = 760)	*P*
Destination from ED			
Intensive care units	162 (8.4%)	86 (11.3%)	
Conventional hospitalization	1,756 (91.2%)	673 (88.6%)	0.036*
Died in ED	8 (0.4%)	1 (0.1%)	
Secondary admission from wards to intensive care units (n = 2,461)	44 (2.5%)	99 (14.7%)	<0.001
Time from ED admission to secondary admission to ICU (days), median [IQR] (n = 114)	1.72 [0.82 – 3.64]	2.76 [0.96 – 4.53]	p=0.312#
All transfers to ICU	206 (10.7%)	185 (24.3%)	< 0.001
Ventilator support			
Invasive mechanical ventilation (n = 2,650)	37 (1.9%)	89 (11.9%)	< 0.001
High-flow nasal cannula (n =2,648)	41 (2.2%)	135 (18.1%)	< 0.001
Non-invasive ventilation (n = 249)	94 (4.9%)	55 (7.4%)	0.633
Length of hospital stay (days) median [IQR] (n=2,365)	6 [2 – 11]	10 [6 – 15]	< 0.001
Decision to withhold or withdraw life-sustaining treatments:			
In ED	90 (4.7%)	53 (7.0%)	0.133
During hospitalization	221 (11.5%)	151 (19.9%)	< 0.002
Death during hospitalization	149 (7.7%)	139 (18.3%)	< 0.001
Death after a decision to withhold or withdraw life-sustaining treatments (n = 288)	105 (70.5%)	96 (69.1%)	0.340
Time from ED admission to death (days) median [IQR] (n = 276)	4.63 [1.70 – 10.84]	8.80 [3.66 – 14.90]	0.127
Outcome after hospital discharge (n = 2,181)			
Return to home	1,382 (84.9%)	397 (71.7%)	< 0.001
Rehabilitation department	245 (15.1%)	157 (28.3%)	< 0.001
Re-hospitalization within 30 days after discharge (n = 2,366)	293 (16.7%)	56 (9.2%)	0.088

*P*-values from multivariate analyses (adjusted for age, gender, body mass index, smoking status, loss of autonomy, chronic respiratory disease, malignancy, bacterial infection, viral co-infection, and oxygen requirement) unless specified

# univariate analysis with Wilcoxon rank-sum test,

* univariate analysis with Fisher’s exact test.

Data are expressed as count (percentage), unless specified otherwise.

*ED*, emergency department; *COVID-19*, coronavirus disease 2019; *IQR*, interquartile range; *ICU*, intensive care unit.

**Table 3:** Univariate and multivariate analyses of factors associated with intensive care unit admission (directly from emergency departments or secondarily from ward).

Variable	Level	OR [95% CI]	*P*-value	OR [95% CI]	*P*-value
COVID-19 positive	Yes	2.69 [2.16; 3.35]		2.24 [1.57; 3.20]	<0.001
Age	≤50	1	<0.001	1	<0.001
	51–65	1.62 [1.15; 2.28]		1.36 [0.83; 2.23]	
	66–80	1.20 [0.87; 1.65]		1.02 [0.64; 1.64]	
	≥81	0.39 [0.27; 0.56]		0.31 [0.18; 0.56]	
Gender	Men	2.26 [1.80; 2.83]	<0.001	1.84 [1.32; 2.60]	<0.001
BMI	<20	1	0.002	1	0.245
	20–25	1.09 [0.71; 1.66]		0.83 [0.49; 1.42]	
	25–30	1.72 [1.14; 2.60]		1.27 [0.75; 2.17]	
	>30	1.65 [1.07; 2.55]		1.04 [0.60; 1.81]	
Current smoker	Yes	1.22 [0.90; 1.66]	0.203	1.25 [0.80; 1.92]	0.324
Loss of autonomy	Yes	0.44 [0.34; 0.58]	<0.001	0.66 [0.43; 1.02]	0.063
Chronic respiratory disease	Yes	1.20 [0.94; 1.54]	0.150	1.01 [0.69; 1.46]	0.950
Immunosuppression	Yes	0.70 [0.47; 1.06]	0.081	-	-
Malignancy	Yes	0.55 [0.22; 1.38]	0.164	0.37 [0.20; 0.65]	<0.001
Bacterial infection	Yes	1.33 [0.96; 1.83]	0.092	1.54 [0.99; 2.36]	0.055
Viral co-infection	Yes	0.66 [0.25; 1.70]	0.361	-	-
Oxygen requirement	Yes	2.95 [2.34; 3.72]	<0.001	4.30 [3.00; 6.17]	<0.001

*COVID-19*, coronavirus disease 2019; *BMI*, body mass index; *OR*, odds ratio; *CI*, confidence interval.

**Table 4:** Univariate and multivariate analyses of factors associated with death during hospitalization.

Variable	Level	OR [95% CI]	P-value	OR [95% CI]	P-value
COVID-19 positive	Yes	2.67 [2.08; 3.42]	<0.001	3.33 [2.02; 5.50]	<0.001
Age	≤50	1	<0.001	1	<0.001
	51–65	4.68 [1.58; 13.80]		1.77 [0.50; 8.28]	
	66–80	11.05 [4.02; 30.39]		3.93 [1.32; 16.94]	
	≥81	22.53 [8.31; 61.09]		6.76 [2.26; 29.25]	
Gender	Men	1.27 [0.99; 1.62]	0.060	1.96 [1.21; 3.24]	0.006
BMI	<20	1	0.127	1	0.313
	20–25	1.05 [0.66; 1.69]		0.74 [0.38; 1.49]	
	25–30	0.69 [0.41; 1.14]		0.51 [0.25; 1.08]	
	>30	0.74 [0.43; 1.28]		0.79 [0.38; 1.70]	
Current smoker	Yes	0.35 [0.20; 0.62]	<0.001	0.68 [0.25; 1.60]	0.399
Loss of autonomy	Yes	2.71 [2.11; 3.47]	<0.001	1.63 [0.98; 2.71]	0.058
Chronic respiratory disease	Yes	0.81 [0.60; 1.11]	0.179	0.90 [0.51; 1.53]	0.696
Immunosuppression	Yes	1.08 [0.72; 1.63]	0.702	-	-
Malignancy	Yes	1.46 [1.02; 2.09]	0.043	1.94 [1.03; 3.52]	0.039
Bacterial infection	Yes	1.72 [1.22; 2.44]	0.003	2.52 [1.49; 4.17]	0.001
Viral co-infection	Yes	0.18 [0.03; 1.36]	0.028	-	-
Oxygen requirement	Yes	3.44 [2.66; 4.45]	<0.001	2.67 [1.66; 4.28]	<0.001

*COVID-19*, coronavirus disease 2019; *BMI*, body mass index; *OR*, odds ratio; *CI*, confidence interval.
