# Supplementary material for: Management and Outcome of COVID-19 Positive and Negative Patients in French Emergency Departments During the First COVID-19 Outbreak: A Prospective Controlled Cohort Study
Source: West J Emerg Med. 2022 Oct 24;23(6):897–906. doi: 10.5811/westjem.2022.7.57135 (PMC9683774; doi:10.5811/westjem.2022.7.57135)
Supplement: Supplementary file 1 [file wjem-23-897-s001.docx]

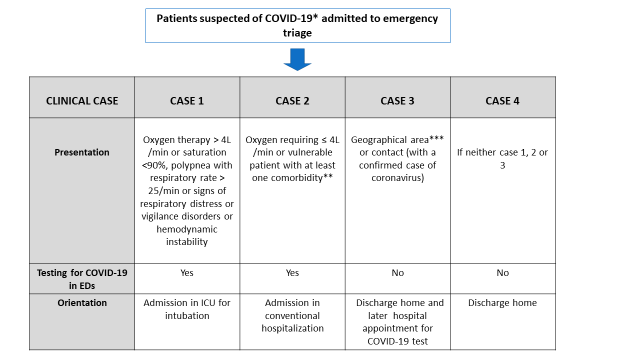


**Supplemental Figure 1 (S1): Triage and orientation of patients suspected COVID 19 in the EDs according to the clinical cases.**

* As the knowledge about the disease grew, we included all patients with at least one of the following symptoms:

- Fever and
- Respiratory symptoms (dyspnea, cough, rhinorrhea/congestion, sore throat..) or
- Other clinical symptoms: nausea or vomiting, anosmia, weakness, diarrhea, myalgia, headache, confusion, abdominal pain, and joint pain.

Patients with another confirmed infectious diagnosis in the EDs such as intra-abdominal, skin and soft tissues, genital and urinary tract infection, meningitis were not considered as suspected patients.

** At least one of the comorbidities among : chronic respiratory disease insufficiency under oxygen therapy, or asthma, or cystic fibrosis, or any chronic pathology that can decompensate during a viral infection, chronic renal failure on dialysis, heart failure stage NYHA III or IV, cirrhosis ≥ stage B, Insulin-dependent or complicated diabetes, immunosuppression (drug, uncontrolled HIV infection, or CD4 <200/mm3 ), organ transplant, hematological malignancy, metastasized cancer, pregnant woman third trimester.

*** Geographical area at risk: China, Singapore, South Korea, Iran, Italy (Lombardy region, Veneto, Emilia Romagna, Piedmont), Haute-Savoie, Oise at the beginning of the study. Later, these data were re-evaluated according to the evolution of the epidemic.
